# Characterization of the clinical relevance and hypoallergenic peptides of the newly evidenced major allergen Hum j 1

**DOI:** 10.3389/fimmu.2025.1588870

**Published:** 2025-06-23

**Authors:** Ya-Li Cheng, Qiong Li, Yong-Shi Yang, Yi-Bo Hou, Zhi-Qiang Xu, Ji-Fu Wei, Jin-Lyu Sun

**Affiliations:** ^1^ Department of Allergy, State Key Laboratory of Complex Severe and Rare Diseases, Peking Union Medical College Hospital, Chinese Academy of Medical Sciences and Peking Union Medical College, Beijing, China; ^2^ Department of Pharmacy, The Affiliated Cancer Hospital of Nanjing Medical University and Jiangsu Cancer Hospital and Jiangsu Institute of Cancer Research, Nanjing, China; ^3^ National Vaccine Innovation Platform, Nanjing Medical University, Nanjing, China; ^4^ Department of Pharmacy, The First Affiliated Hospital of Nanjing Medical University, Nanjing, China

**Keywords:** *Humulus japonicus* pollen, Hum j 1, allergic asthma, hypoallergenic peptides, allergy diagnosis

## Abstract

**Background:**

*Humulus japonicus* (HJ) pollen is a predominant autumn allergen in northern China. Two decades ago, a 10 kDa protein termed Hum j 1 was proposed as a major allergen, but its uncertainty hindered its clinical application. This study aims to investigate the clinical relevance of Hum j 1 and screen hypoallergenic peptides for potential application in molecular diagnosis and immunotherapy.

**Methods:**

Serum samples from 93 HJ pollen-allergic patients were analyzed for IgE reactivity against recombinant Hum j 1 (rHum j 1). We evaluated correlations between IgE responses to rHum j 1 and HJ pollen crude extracts using Spearman’s rank correlation analysis. The association between clinical symptoms and Hum j 1-IgE positivity was evaluated by group comparisons and multivariable analyses. Allergenicity of Hum j 1 was further investigated by immunoblotting and basophil activation tests. Six KLH-coupled peptides (21–25 amino acids) spanning the complete Hum j 1 sequence were synthesized to assess hypoallergenicity and IgG-mediated inhibitory effects against allergen-specific IgE binding using a murine passive immunization model.

**Results:**

rHum j 1 demonstrated IgE reactivity in 74.2% (69/93) of the patients and induced significant basophil activation. rHum j 1-specific IgE levels showed a moderate positive correlation with crude extract-specific IgE levels (Spearman’s ρ = 0.529, *p* < 0.0001). Patients with allergic rhinitis complicated by asthma had significantly higher levels of Hum j 1-sIgE (*p* = 0.014). We found a significant association between Hum j 1 sensitization and asthma in the multivariate analysis [odds ratio (OR) = 3.98, 95% confidence interval (CI): 1.2–13.0, *p* = 0.02], with Hum j 1-sensitized patients showing higher asthma prevalence compared to non-sensitized individuals (46% vs. 17%, *p* = 0.010). All six peptides showed significantly reduced IgE reactivity (*p* < 0.0001) and minimal basophil activation. Immunized mice produced high-titer IgG antibodies that inhibited patient IgE binding to rHum j 1 by 22.09%–64.61%.

**Conclusions:**

Hum j 1 could enhance the sensitivity of HJ pollen crude extract-based IgE assays. IgE reactivity to Hum j 1 was more frequently associated with allergic asthma. The hypoallergenic linear peptides of Hum j 1 laid the foundation for the development of a molecular vaccine for allergen-specific immunotherapy. These findings would contribute to developing diagnostic and therapeutic strategies for HJ pollinosis.

## 1 Introduction

Pollen allergy affects 10%–20% of the global population (1). Inhalation of allergenic pollen is one of the most important factors causing rhinitis, conjunctivitis, and even allergic asthma a (2–4). To make matters worse, in real-world clinical practice, we sometimes encounter patients with pollen allergy suffering from severe extra-respiratory symptoms, including skin, gastrointestinal, or flu-like symptoms in relation to exposure to sensitized pollen (5). In recent years, research shows that the incidence of pollen allergy in China has increased from 5% to 17.8%, with a growing trend (6, 7). *Humulus japonicus*, belonging to the Cannabaceae family, is an annual or perennial herbaceous plant with creeping stems and spiny hair (6). *H. japonicus* flowers from July to September and is distributed in most areas of China and other Asian countries, including Japan and Korea (2, 5, 8). HJ pollen is one of the most important autumn pollen allergens associated with allergic asthma and seasonal rhinitis (9). In summer and autumn, the content of HJ pollen in the air in most cities of northern China was second only to *Artemisia*, and even higher in some areas (10).

Research on major allergens is pivotal for understanding allergic sensitization mechanisms and developing targeted diagnostic and therapeutic tools (11). Major allergens account for over 50% of IgE reactivity in sensitized populations, serving as molecular targets for component-resolved diagnosis (CRD) and immunotherapy (12, 13). For example, a number of well-known representative major inhalant pollen allergens, such as Bet v 1 in birch pollen, Amb a 1 in ragweed, Art v 1 in mugwort, and Phl p 5 in timothy grass pollen, have been well elucidated (12). Recent studies highlight that CRD based on major allergens significantly improves diagnostic specificity by distinguishing genuine sensitization from cross-reactivity (14), thereby optimizing patient-specific management strategies. Furthermore, recombinant major allergens have revolutionized immunotherapy development, enabling safer and more effective formulations, such as hypoallergenic derivatives and T-cell epitope vaccines. However, as one of the most important autumn allergenic pollens in Asian countries, the major allergens of HJ pollen have been less well studied.

Previous studies have reported several allergens in *Humulus* pollen, of which a 10 kDa allergen contributed 88% of the specific IgE against HJ pollen extracts and was named Hum j 1 by the World Health Organization and International Union of Immunological Societies (WHO/IUIS) Allergen Nomenclature Sub-Committee (https://www.allergen.org/) (15). Although Hum j 1 was listed in the WHO-IUIS database, the molecular features of Hum j 1 remain unresolved. Reproducibility challenges have been widely documented, with independent laboratories failing to consistently detect the 10 kDa protein using 2-dimensional gel analysis (16, 17). Moreover, a critical mismatch exists between the determined N-terminal sequence (DNXFENGMKAXTSLYDXKYQ) (15) of the natural molecule and the amino acid sequence in the WHO-IUIS database and GenBank (Accession AAP94213), leading to uncertainties regarding the allergen’s actual molecular identity. Fortunately, our group has successfully cloned the cDNA encoding Hum j 1 based on the results from mass spectrometry of purified natural Hum j 1 and transcriptomics of HJ pollen (unpublished data). This enables us to prepare the recombinant molecule and assess its potential clinical applications.

In this study, we conducted an exploratory analysis of the association between Hum j 1-specific IgE reactivity and clinical phenotypes in a well-characterized cohort of *H. japonicus* pollen-allergic patients, with a focus on its diagnostic utility for identifying asthma-prone subgroups. Furthermore, we designed six overlapping peptides covering the entire amino acid sequence of Hum j 1, and after coupling with keyhole limpet hemocyanin (KLH), their hypoallergenicity and the ability to induce blocking IgG against the specific IgE were evaluated. Furthermore, we designed six KLH-conjugated overlapping peptides spanning the full-length sequence of Hum j 1 and comprehensively assessed their hypoallergenic properties and capacity to induce IgG antibodies capable of blocking IgE-allergen interactions. Collectively, this work aims to (1) resolve the molecular basis of HJ pollen allergy through component-resolved diagnostics and (2) establish a proof-of-concept platform for epitope-targeted immunotherapy, addressing critical gaps in both risk stratification and therapeutic innovation for pollen-driven allergic comorbidities.

## 2 Materials and methods

### 2.1 Patient serum samples

A total of 93 patients with a *H. japonicus* allergy were enrolled in the Allergy Department of the Peking Union Medical College Hospital (PUMCH). All the enrolled patients suffered from allergic rhinitis, with or without other allergic diseases. All the patients had clear allergic symptoms in autumn. They had positive HJ pollen-specific IgE results (≥ 0.7 kUA/L) measured using the ImmunoCAP system (Thermo Fisher Scientific, Uppsala, Sweden) and/or positive skin test to HJ pollen extract. The detailed clinical information for these patients is listed in **Supplementary Table S1**. Sera from healthy individuals without allergic symptoms were enrolled as negative controls. The present study was approved by the Ethical Committee of Peking Union Medical College Hospital (I-23PJ1573).

### 2.2 The recombinant Hum j 1 protein and Hum j 1-derived KLH-coupling peptides

The recombinant Hum j 1 was prepared following protocols established in a separate manuscript (unpublished) that comprehensively details the allergen’s identification, molecular cloning, and characterization. Endotoxins were effectively removed during anion-exchange chromatography (18) by leveraging the strong negative charge of endotoxins, which retained them on the column while rHum j 1 was eluted. Residual endotoxin levels were quantified via a chromogenic lyophilized amebocyte lysate (LAL) assay according to the manufacturer’s instructions, demonstrating compliance with FDA standards (<0.5 EU/mL).

Six overlapping peptides (P1-P6) spanning the full-length Hum j 1 sequence were designed based on its cloned sequence, with 21–25 amino acid lengths and 6–12 residue overlaps. Peptides were synthesized via Fmoc solid-phase synthesis (Sangon Biotech, Shanghai, China), purified to >95% purity using high-performance liquid chromatography (HPLC) on a Shimadzu Shim-pack GIST C18 column (250 × 4.6 mm, 5 μm), and verified by Matrix-Assisted Laser Desorption/Ionization Time-of-Flight (MALDI-TOF) mass spectrometry (detailed characteristics in **Supplementary Table S2**). Each peptide contained a terminal cysteine residue (C- or N-terminus) for maleimide-mediated covalent coupling to KLH. KLH-conjugated peptides were dissolved in PBS (pH 7.4), stored at -20°C, and quantified using a Micro BCA Protein Assay Kit.

### 2.3 The IgE binding capacity of the recombinant Hum j 1 and Hum j 1-derived peptides

IgE binding to Hum j 1 was determined in patients’ sera by ELISA according to our previous study (19). A 96-well microplate was pre-coated with 100 µL per well of 10 µg/mL rHum j 1 in carbonate buffer (pH 9.6) and incubated at 4°C overnight. Furthermore, the 93 serum samples (diluted 1:10) included in the study were added to the plate (100 µL/well) and incubated at 37°C for 2 h. Then, 100 µL of horseradish peroxidase (HRP)-conjugated goat anti-human IgE (KPL, Maryland, USA; Catalog No. 5220–0329) (diluted 1:2,500 in PBST) was added for another 1-hour incubation. Subsequently, the color was developed using tetramethylbenzidine (TMB) substrate (Beyotime, Shanghai, China). The reaction was then stopped by adding 50 μL 2 M H_2_SO_4_, and the absorbance was measured at 450 nm using Multiskan GO (Thermo Fisher Scientific, Massachusetts, USA). The cut-off values were presented as the mean optical density (OD) value + 3 standard deviations (SD) of the negative controls.

For Hum j 1-derived peptides, serum samples from 38 Hum j 1-sIgE positive patients were selected for ELISA as previously described (29). To prevent the formation of disulfide bonds between peptide segments, we added an appropriate amount of dithiothreitol. Briefly, the levels of six KLH-peptides specific IgE antibodies from 38 Hum j 1-sIgE positive patients (diluted 1:5) were measured.

For further IgE immunoblot analysis of Hum j 1, we selected the serum samples of patients with the top five OD values in the ELISA experiment. HJ pollen extract or the rHum j 1 was separated on FuturePAGE Precast Protein Gel and then transferred onto 0.22 µm polyvinylene difluoride (PVDF) membranes. PVDF membranes were then blocked with 5% (w/v) skimmed milk in PBS for 2 h at room temperature. Subsequently, patient serum samples (diluted 1:10 in PBS) were used as the primary antibody to incubate the membranes overnight at 4°C. After washing three times with PBST, the membrane was incubated with HRP-conjugated goat anti-human IgE antibody (diluted 1:5,000 in PBST) for 1 h at room temperature with shaking. Positive protein bands were visualized by Immobilon™ Western HRP Substrate Luminol Reagent (Merck Millipore, Massachusetts, USA) using a chemiluminescent imaging system.

### 2.4 The association between IgE recognition of Hum j 1 and clinical phenotypes

In order to study possible associations between IgE recognition of Hum j 1 and clinical symptoms, we used sera from 93 HJ pollen-allergic patients who had been characterized for IgE reactivity to Hum j 1 by ELISA. In the cohort of allergic rhinitis patients (**Supplementary Table S1**), allergists assessed their comorbidities based on detailed medical records from their initial allergy department visit, including documented presentations of conjunctivitis (seasonal itching, redness, tearing), asthma, eczema, atopic dermatitis, or urticaria.

### 2.5 Allergenic activity of human serum with the Hum j 1 and Hum j 1-derived peptides

Basophil activation tests (BATs), based on stimulation of whole blood cells from HJ pollen-allergic patients and measuring CD63 activation of basophils using CCR3 as a basophil marker by flow cytometry (Flow CAST^®^, BÜHLMANN Laboratories AG, Schönenbuch, Switzerland) according to the manufacturer’s instructions, were performed with Hum j 1 and Hum j 1-derived KLH-peptides. Briefly, the allergen or peptides mimic the *in vivo* reaction where specific IgE bound to the cellular surface are bridged by the culprit allergen and activate an intracellular signaling cascade, leading to the activation of the basophil. Basophils were stimulated with Hum j 1 protein or Hum j 1-derived peptides (final concentration, 10 µg/mL). The stimulation was performed for 15 min at 37°C in a stimulation buffer included in the kit, containing calcium and IL3, and a staining reagent including anti-CD63-FITC and anti-CCR3-PE antibodies. A flow cytometry analysis was performed to calculate the percentage of activated basophils, determined as the percentage of CD63-positive cells in the population of CCR3^pos^/SSC^low^ according to the manufacturer’s instructions. Basal activation and positive control activation by an anti-FcϵRI receptor monoclonal antibody included in the kit were checked for each of the independent basophil donors. BATs were considered positive according to the technical cutoff of CD63^pos^ basophils ≥ 15% in the Flow CAST^®^ test, as indicated by the manufacturer.

### 2.6 Mouse immunization and determination of Hum j 1- and peptide-specific IgG antibody levels

Mouse antibodies specific for Hum j 1 and Hum j 1-derived peptides were generated through triplicate immunizations at monthly intervals using 200μg of rHum j 1 or KLH-coupled peptides (KLH-P1 to KLH-P6 and KLH-Pmix), a dose consistent with established murine immunization protocols for antibody production (20). Initial immunization employed Freund’s complete adjuvant, with subsequent boosts using incomplete Freund’s adjuvant. KLH-Pmix immunizations utilized equal amounts of all six peptides.

Hum j 1- and peptide-specific IgG, IgG_1_, IgG_2a_, and IgG_2b_ antibody levels were measured by ELISA. Briefly, ELISA plates were coated with 10 μg/mL of rHum j 1, KLH-coupled peptides (P1–P6), or Pmix (100 µL) and incubated overnight at 4°C. Following blocking with 1% BSA in PBST, serum samples from individual mice were added in duplicate (100 μL/well) and incubated for 2 h at 37°C. Serial five-fold dilutions were prepared from 5 × 10^−^² to 3.90625 × 10^−7^ for IgG quantification, while fixed 1:50 dilutions were used for IgG_1_, IgG_2a_, and IgG_2b_ detection. After washing, bound antibodies were detected using HRP-conjugated goat anti-mouse IgG antibodies (SouthernBiotech) at a 1:2,500 dilution, followed by TMB substrate development.

### 2.7 Competition assays determining the inhibition of allergic patients’ IgE binding to rHum j 1 by specific mouse antibodies

IgE inhibition ELISAs were performed as described (21, 22), using rHum j 1 or BSA as a control coated on the ELISA plates in carbonate buffer (pH 9.6) at 4°C overnight. The plates were then preincubated with mouse anti-rHum j 1; anti-KLH coupled P1, P2, P3, P4, P5, and P6; and Pmix antisera or the corresponding pre-immune sera overnight at 4°C, respectively. Mouse sera from CFA-immunized mice were diluted to a ratio of 1:20. After washing, the plates were incubated with sera from HJ pollen-allergic patients (1:10 in PBST) for 2h at 37°C. Bound human IgE Abs were detected with 1:2,500 diluted HRP-coupled goat anti-human IgE Abs. The percentages of inhibition of IgE binding to Hum j 1 due to pre-incubation with mouse anti-peptide IS were determined by comparing the OD values obtained upon pre-incubation with the pre-immune sera (OD_P_) or peptide-specific immune sera (OD_I_) after subtracting the background values determined for BSA-coated wells (OD_B_) according to the following formula: 100-(OD_I_-OD_B_)/(OD_P_-OD_B_) × 100.

### 2.8 Statistical analysis

All graphs were performed using Origin software (Origin Lab Corporation, Northampton, MA, US). Descriptive parameters, such as means and standard deviations (SD), and frequencies and percentages for categorical data, were calculated. The two-sample t-test was used to evaluate the continuous variable. The association between specific IgE reactivity and clinical symptoms was analyzed by applying Fisher’s exact probability test. The Spearman rank test was used to assess correlations. All analyses were performed using SPSS 26.0 software (IBM, Armonk, NY). A *p-*value < 0.05 was considered statistically significant (*p < 0.05, **p < 0.01, ***p < 0.001).

## 3 Results

### 3.1 Hum j 1 is a major allergen in Chinese HJ-allergic patients

**Figure 1:**
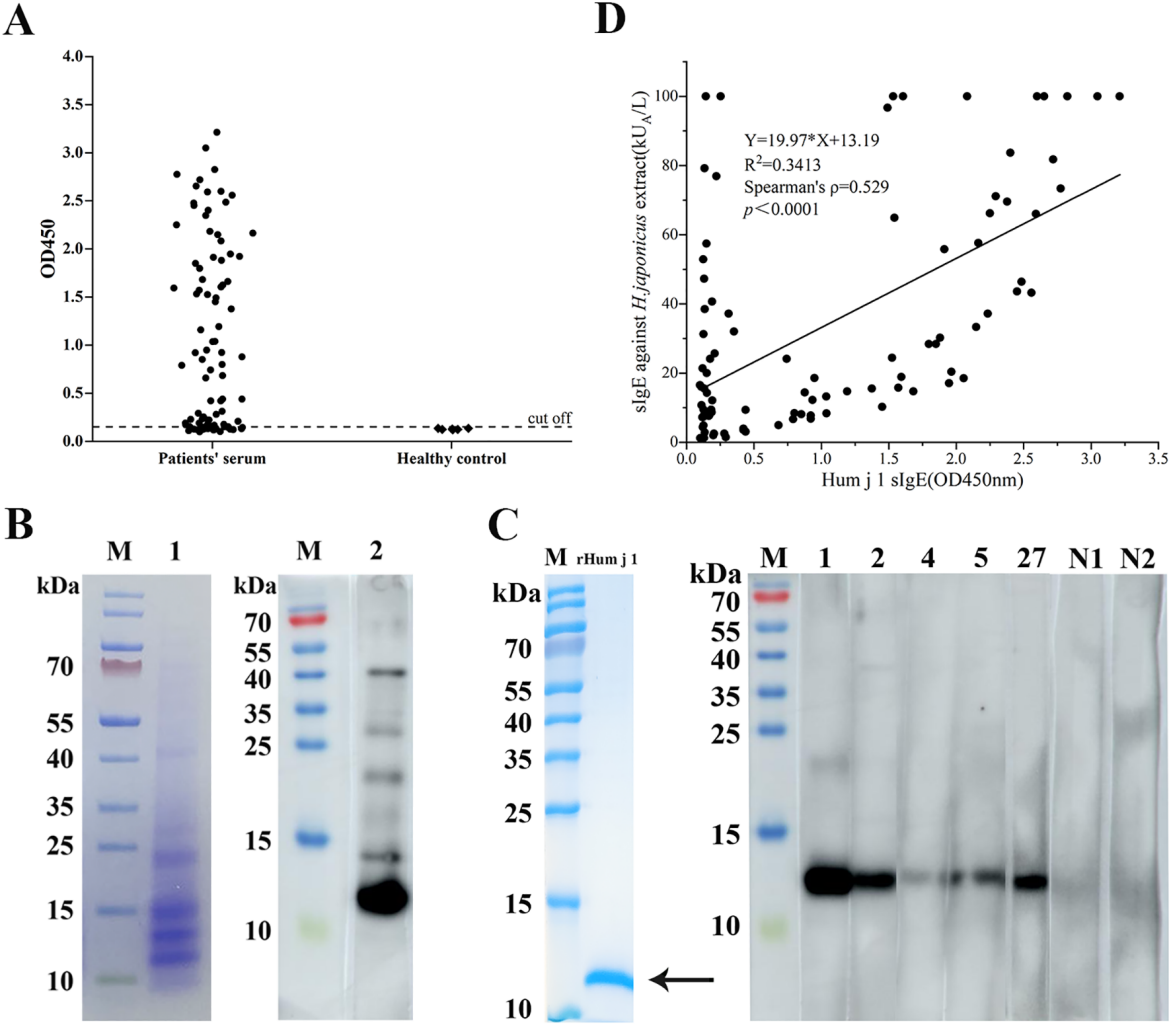
IgE reactivity of the recombinant Hum j 1. **(A)** ELISA of 93 HJ pollen-allergic patients and 6 controls (cutoff: mean negative +3SD). Data: mean ± SD (triplicates). **(B)** Left: SDS-PAGE of HJ pollen extract (Coomassie staining). Right: Western blot probed with pooled sera from 5 patients. **(C)** Purified rHum j 1 (arrow, Coomassie-stained) probed with top 5 ELISA-positive and 2 negative sera. **(D)** Correlation between rHum j 1 IgE (OD450) and HJ pollen extract sIgE in patients (n=93).

The coding regions of Hum j 1 cDNA were expressed in *Escherichia coli* BL-21 (DE3). As shown in **Figure 1C**, there is a single band in SDS-PAGE with an approximate molecular weight of 10 kDa. The prevalence of rHum j 1 sensitization among HJ pollen-allergic patients (n = 93) was evaluated using allergen-specific ELISA. As shown in **Figure 1A**, 69 out of 93 patients (74.2%) displayed positive IgE-reactivity to rHum j 1. Further, as shown in the IgE immunoblot experiments (**Figure 1B**), a 10-kDa protein component of HJ pollen extract showed the most potent IgE reaction. Meanwhile, among the five sera with strong IgE reactivity in ELISA, except for serum 1, which showed a strong IgE binding band at 10 kDa, the other four sera showed weakened IgE binding, especially in sera 4 and 5 (**Figure 1C**).

### 3.2 Hum j 1 improves extract-based IgE diagnostic sensitivity

We performed Spearman’s correlation analysis to assess the relationship between specific IgE levels against HJ pollen crude extract (ImmunoCAP) and rHum j 1 (ELISA OD values) in 93 HJ pollen-allergic patients, given the non-normal distribution of both datasets. A statistically significant moderate positive correlation was observed (Spearman’s ρ = 0.529, *p* < 0.0001; **Figure 1D**), indicating that elevated Hum j 1-sIgE levels correlate with heightened IgE reactivity to HJ pollen extract. Notably, two distinct outlier subgroups were identified. (i) In the y-axis cohort (n = 12), the patients exhibited high HJ pollen extract-sIgE but low Hum j 1 reactivity. This divergence may reflect the presence of additional allergenic components in HJ pollen or cross-reactivity from polysensitization, as 10/12 (83.3%) were sensitized to ≥ 2 seasonal pollens. (ii) In the below-the-correlation-line cohort (n = 32), the patients showed low HJ pollen extract-sIgE but high Hum j 1 reactivity, suggesting Hum j 1’s superior diagnostic sensitivity. Strikingly, 53.1% (17/32) of this subgroup had comorbid asthma, reinforcing Hum j 1’s clinical relevance in high-risk phenotypes. In contrast, 19 patients aligned closely with the correlation line demonstrated strong concordance between HJ pollen extract- and Hum j 1-specific IgE levels, further validating Hum j 1 as a dominant allergenic component.

### 3.3 Hum j 1 induces high basophil activation

**Figure 2:**
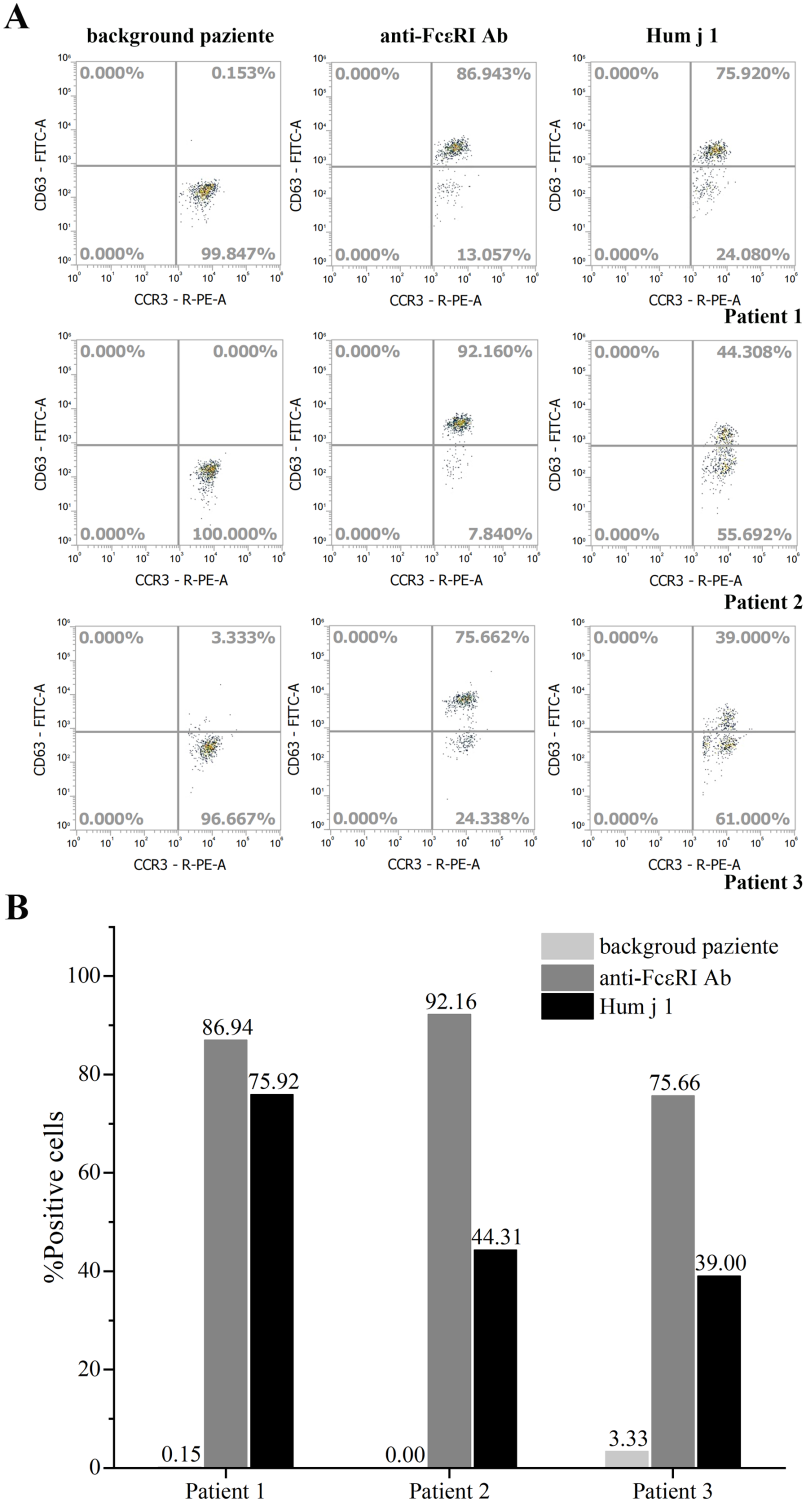
Basophil activation test of rHum j 1. **(A)** Allergenic activity of rHum j 1. Representative flow cytometry plots of basophils from 3 different Humulus japonicus allergic patients. Basophil activation was assessed by monitoring the proportion of CCR3 and CD63 positive cells. **(B)** The calculated proportion (%) of CCR3 and CD63 positive cells after stimulated with either anti-FcεRI, buffer control or the rHum j 1, respectively.

The allergenic activity of Hum j 1 was studied in basophil activation experiments by measuring the proportion (%) of CD63-positive and CCR3-positive cells. Compared with the activation of 86.94%, 92.16%, and 75.66% of basophils treated with anti-FcϵRI mAb in three patients, Hum j 1 induced half or even equivalent activation of basophils in the corresponding group, with activation rates of 75.92%, 44.31%, and 39.00% in basophils, respectively (**Figures 2A, B**). According to the manufacturer’s instructions, the three BATs for Hum j 1 were considered positive (percentage of CD63^+^ basophils ≥ 15%). The results indicated that Hum j 1 has a strong basophil-activating capacity.

### 3.4 Patients with allergic rhinitis combined with asthma have higher Hum j 1 specific IgE-binding absorbance values

We analyzed the clinical characteristics of 93 patients with an allergy to HJ pollen included in our study. They were aged 3–62 years (mean age 28.4 ± 16.1 years), with 43 male and 50 female patients (**Supplementary Table S1**). According to the classification of other concurrent allergic diseases, there were 63 participants with allergic rhinitis complicated with conjunctivitis, 36 participants with allergic rhinitis complicated with asthma, 19 participants with allergic rhinitis combined with dermatitis or eczema, and 10 participants with allergic rhinitis complicated with urticaria (**Supplementary Table S1**).

**Figure 3:**
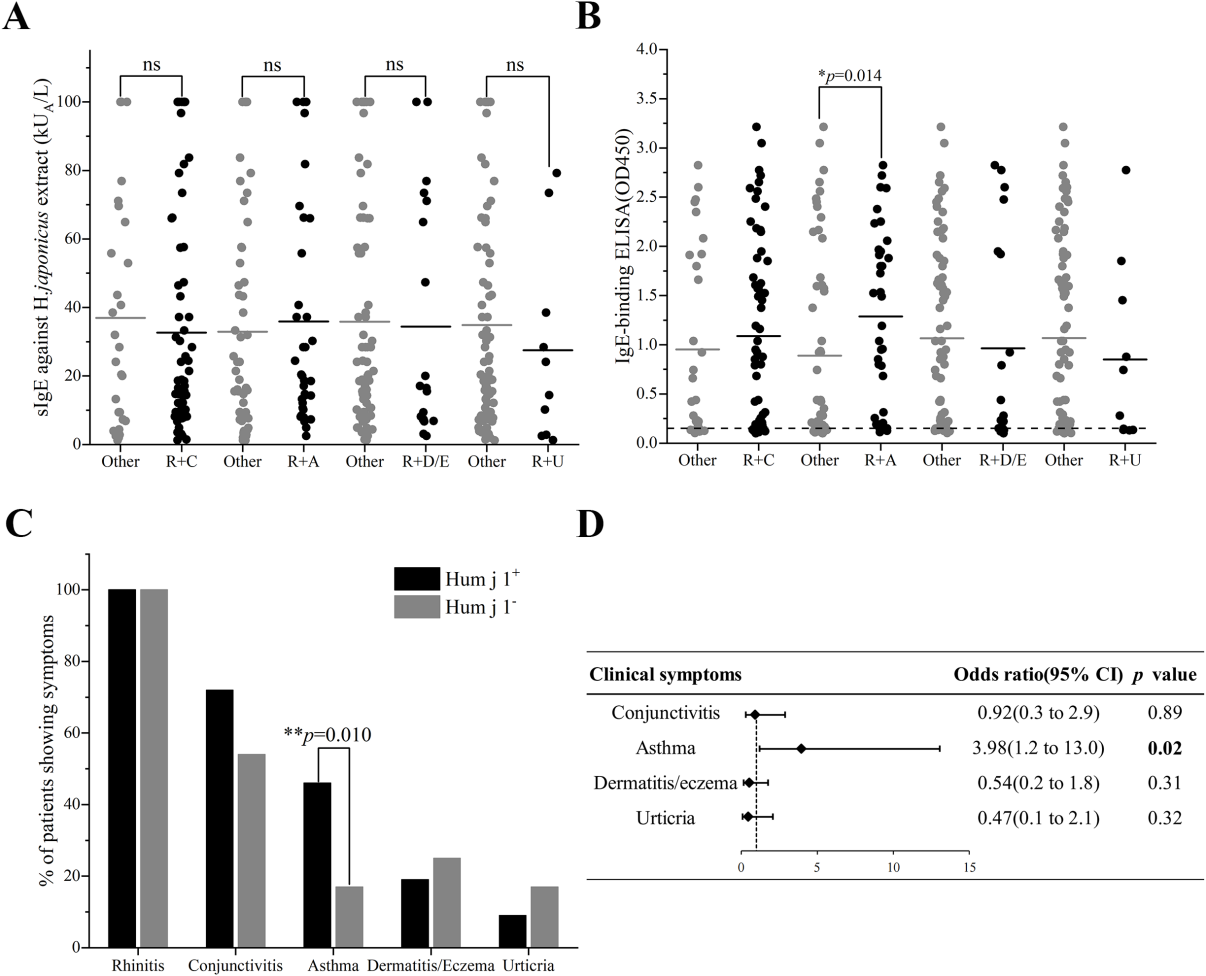
Clinical relevance of Hum j 1 sensitization. **(A)** HJ pollen sIgE (ImmunoCAP) or **(B)** Hum j 1-sIgE (OD, triplicates) in patients with rhinitis plus conjunctivitis (R+C), asthma (R+A), dermatitis/eczema (R+D/E), or urticaria (R+U). **(C)** Symptom prevalence (%) in Hum j 1-sensitized (red) vs. non-sensitized (black). **(D)** Multivariate analysis of clinical symptoms and Hum j 1 sensitization. Differences of HJ pollen sIgE value (ImmunoCAP) or Hum j 1-sIgE absorbance value (ELISA) between groups were calculated by the Kruskal-Wallis test. Frequencies of IgE-binding between groups were analyzed by chi-square test. *p < 0.05, **p < 0.01.

We analyzed the relationship between the clinical phenotypes of the patients and the sIgE titers of HJ pollen crude extract or Hum j 1, respectively. After comparing the differences in sIgE levels of HJ pollen crude extract in allergic rhinitis with/without other clinical symptoms, there was no significant difference between the groups (**Figure 3A**). However, we found that the Hum j 1 sIgE levels in the patients with allergic rhinitis co-existing with asthma were significantly higher than those in patients without asthma {median of 1.321 [interquartile range (IQR); 0.298–1.951] vs. median of 0.291 (IQR; 0.133–1.594), *p* = 0.014} (**Figure 3B**).

### 3.5 Allergic patients with asthma have a higher frequency of sensitization to Hum j 1

In exploratory analyses evaluating the clinical relevance of Hum j 1 sensitization, we observed a significant association between Hum j 1-sensitization and allergic rhinitis combined with asthma in the multivariate analysis [odds ratio (OR) 3.98, 95% confidence interval (CI):1.2–13.0, *p* = 0.02] (**Figure 3D**). Asthma prevalence was elevated in Hum j 1-sensitized patients (46%, 32/69) vs. non-sensitized individuals (17%, 4/24; *p* = 0.010) (**Figure 3C**), where the non-sensitized subgroup contained only four asthmatic patients among 24 total subjects. No relevant differences were found between Hum j 1-sensitized patients and non-Hum j 1-sensitized patients regarding other HJ pollen allergy-related symptoms, including allergic conjunctivitis (70% vs. 59%, *p* = 0.35), dermatitis or eczema (18% vs. 27%, *p* = 0.41), and urticaria (8% vs. 18%, *p* = 0.29) (**Figure 3C**).

### 3.6 Six synthetic peptides were hypoallergenic peptides

**Figure 4:**
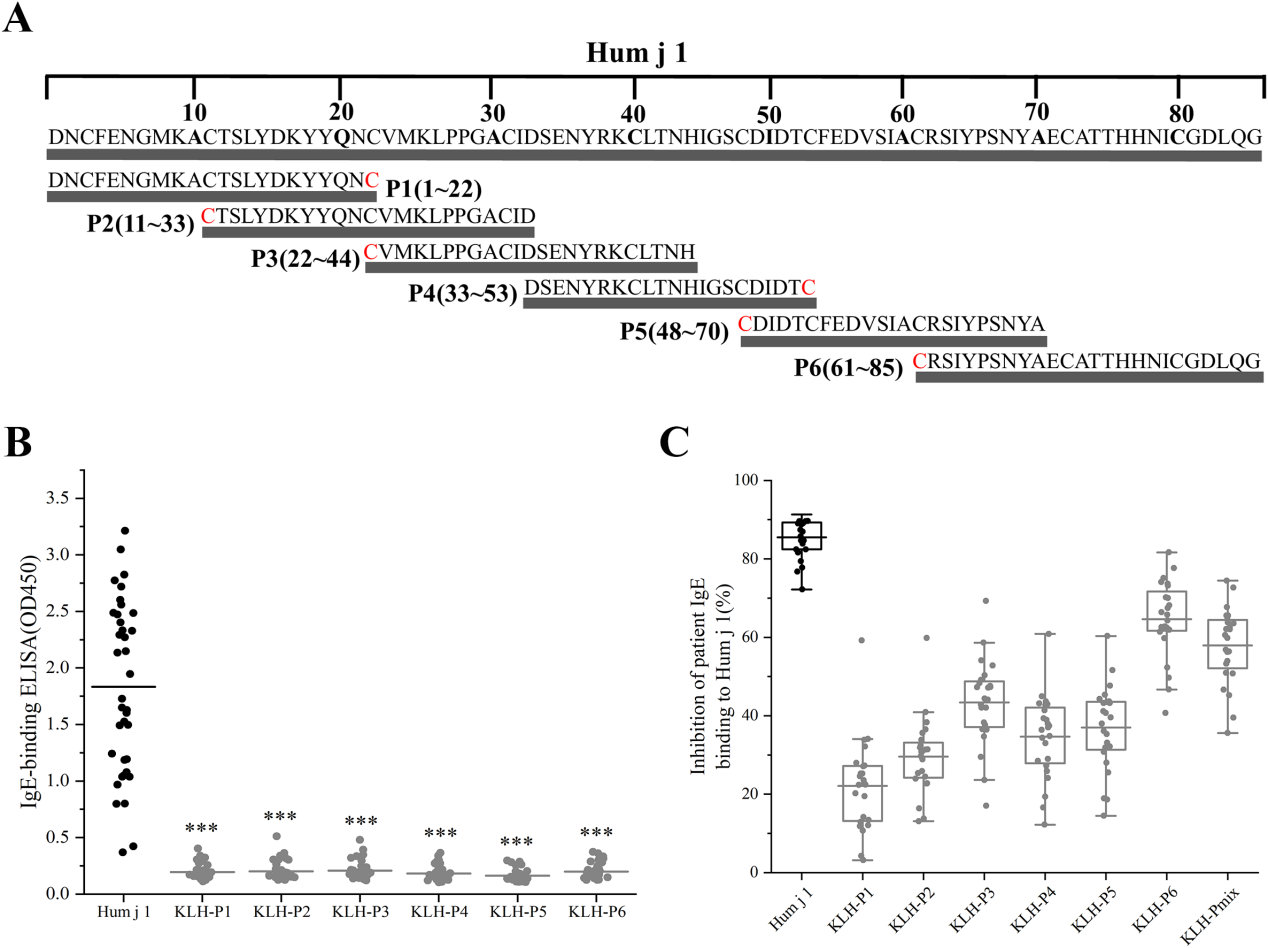
Hum j 1 derived hypoallergenic peptide screening. **(A)** Schematic of Hum j 1-derived peptides (P1-P6) with Cys termini (red). **(B)** IgE reactivity of KLH-conjugated peptides in Hum j 1 sensitized patients (n=38). **(C)** Percentages inhibition of patients (n = 24) IgE binding to rHum j 1 after preincubation of rHum j 1 with mouse anti-peptide/anti–Hum j 1 antibodies.

We synthesized six overlapping peptides, with a length of 21–25 aa, which allowed us to cover the sequences of the important HJ pollen allergen-Hum j 1, and conjugated them with KLH for subsequent analysis (**Figure 4A**; **Supplementary Table S2**). As shown in **Figure 4B**, compared to Hum j 1, the IgE reactivity of KLH-P1–P6 was significantly reduced (1.83 vs. 0.20, 0.20, 0.21, 0.18, 0.16, and 0.20, *p* < 0.001) (**Figure 4B**).

To evaluate the allergenic activity of KLH-conjugated peptides, we performed BATs using whole blood from three HJ pollen-allergic donors. All six KLH-peptides demonstrated no basophil activation, with the percentage of CD63+ basophils below the 15% clinical threshold according to the manufacturer’s instructions (**Supplementary Figure S1**). Specifically, KLH-P1 exhibited activation rates of 8.46% (Donor 1), 0.49% (Donor 2), and 5.50% (Donor 3), while KLH-P2 showed rates of 8.62%, 0.31%, and 3.00%, respectively. The highest observed activation occurred with KLH-P3 in Donor 1 (10.00%), contrasting with the minimal responses in Donors 2 (0.77%) and 3 (2.32%). The remaining peptides demonstrated even lower reactivity: KLH-P4 with 6.83%, 0.31%, and 1.00%; KLH-P5 with 0.62%, 0.92%, and 2.00%; and KLH-P6 with 6.92%, 0.00%, and 4.83%. The consistent low activation of all KLH-peptides among the three donors strongly supports the hypoallergenic profile of all peptide candidates.

### 3.7 KLH-P6 and KLH-Pmix induce high levels of Hum j 1-specific IgG Abs in mice that inhibit patients’ IgE binding to Hum j 1

Mouse sera obtained after immunization with rHum j 1 or the KLH-coupled peptides using Freund’s adjuvant were tested for IgG reactivity by ELISA. All KLH-coupled peptides induced high levels of specific IgG titers against the corresponding immunogens, revealing full saturation of binding at low dilutions (1:500), whereas the mouse pre-immune sera did not show relevant IgG reactivity (**Supplementary Figure S2**). Moreover, IS raised against KLH-P6 showed approximately five-fold stronger reactivity as compared with IS raised against the full-length rHum j 1 and other remaining peptides. The IgG subclass profiling demonstrated differential immunogenicity among the KLH-conjugated peptides (**Supplementary Figure S3**). KLH-P3 and KLH-P6 elicited robust IgG_1_ titers comparable to wild-type Hum j 1, while inducing significantly elevated IgG_2a_ and IgG_2b_ responses relative to the wild-type allergen. Notably, KLH-P6 showed the strongest IgG_2a_ induction among all the candidates. KLH-P5 exhibited moderate IgG_1_ production, which was lower than wild-type levels, with low IgG_2a_/IgG_2b_ reactivity. Strikingly, the KLH-Pmix formulation generated the highest IgG_2b_ titers recorded and moderate IgG1 levels.

We then tested whether IgG Abs induced by immunization with the KLH-coupled peptides could inhibit HJ pollen-allergic patients’ IgE binding to Hum j 1 in ELISA competition assays, using sera from 24 HJ pollen-allergic patients (**Supplementary Table S3**). **Figure 4C** shows that inhibition of patients’ IgE binding to rHum j 1 after preincubation with the respective mouse anti-rHum j 1 IS occurred in all patients, amounting to 85.50% ± 4.95% (mean ± SD; range, 72.20%–91.31%). Moreover, peptide-specific IS anti-KLH-P6 and anti-KLH-Pmix revealed comparable IgE-blocking activity (**Figure 4C**; **Supplementary Table S3**). Specifically, IgE binding to rHum j 1 was inhibited on preincubation with mouse anti-KLH-P6 IS (**Figure 4C**), with mean inhibition values of 64.61% ± 9.63% (range of 40.72%–81.67%). Similar to anti-KLH-P6 IS, mouse anti-KLH-Pmix IS significantly inhibited (57.96% ± 9.47% inhibition; range of 35.59%–74.44%) IgE binding to rHum j 1. Comparatively, the inhibition of patients’ IgE binding to Hum j 1 with mouse anti-KLH-P1, P2, P3, P4, and P5 Abs was considerably lower, with inhibition ranging from 22.09% to 43.39% (**Supplementary Table S3**). Therefore, KLH-P6 and KLH-Pmix are candidate vaccines for immunotherapy.

## 4 Discussion

*H. japonicus* is one of the most potent and widespread sources of pollen allergens in East Asia, causing pollinosis and severe respiratory allergy in sensitized patients (9, 17). Even more significantly, the symptoms of asthma triggered by HJ pollen are reportedly more serious than those induced by *Artemisia* pollen (23). However, there is still limited research on the major components of HJ pollen. A valuable major HJ pollen allergen, Hum j 1, was reported early on, but the lack of accurate and solid evidence has hindered its development. Our research group unexpectedly identified accurate sequence information of Hum j 1 through transcriptomics and mass spectrometry (the data is currently unpublished). Possible associations between IgE responses to Hum j 1 and clinical phenotypes have not yet been evaluated. This study aimed to establish the sensitization patterns of Hum j 1 and its possible associations with clinical phenotypes in China. In addition, we used the overlapping peptides approach to convert Hum j 1 into a hypoallergenic vaccine. This approach is based on the identification of peptides derived from the IgE binding sites of allergens, which lack IgE reactivity and allergenic activity, as well as exhibiting the inhibition of HJ pollen-allergic patients’ IgE binding to Hum j 1.

In the present study, the IgE reactivity of Hum j 1 was determined in sera from clinically well-characterized HJ pollen*-*allergic patients using ELISA. Hum j 1 was recognized by >70% of the HJ pollen-allergic patients, similar to a previous report (15). There are many studies on the sensitization rates of other well-known representative major pollen allergens. For instance, Bet v 1 is the key molecule responsible for birch pollen allergy, with IgE-binding frequencies >95% (24). Similarly, more than 90% of ragweed-allergic patients showed IgE binding to Amb a 1, the major allergen of ragweed pollen (25). In this study, although the IgE reactivity of recombinant Hum j 1 was 74.2% (69/93), the ELISA absorbance value of rHum j 1 was extremely high, indicating the strong IgE reactivity capacity of rHum j 1. More importantly, Hum j 1 exhibited high allergenic activity, as determined by its ability to induce IgE-dependent basophil activation. Compared with the determination of sIgE in serum, a BAT reflects a functional response and has become a widely used measure of allergic activity (26). In the present study, Hum j 1 caused significant upregulation in the proportion (%) of CCR3 and CD63 double-positive cells, further indicating that it is a clinically relevant and biologically active allergen in HJ pollen. Taken together, Hum j 1 must be considered an essential component in vaccines for immunotherapy for HJ pollen allergy.

A statistically significant positive correlation was observed between rHum j 1-specific IgE reactivity and HJ pollen extract-specific IgE levels (Spearman’s ρ = 0.529, *p* < 0.0001), suggesting that Hum j 1 may serve as a potential diagnostic marker for HJ pollen allergy. By reviewing the medical records of the 93 enrolled patients, we further analyzed the correlation between Hum j 1 sensitization and clinical phenotypes. In all the tested patients, the HJ pollen extract IgE values in the allergic asthma group were slightly higher than the non-asthmatic group, but not significantly, while Hum j 1 showed a statistically significant difference in patients with allergic rhinitis combined with asthma. Furthermore, our analysis demonstrated significantly higher allergic asthma prevalence in Hum j 1-sensitized patients compared to non-sensitized individuals. When integrating these sensitization patterns with the Hum j 1-sIgE level assessments, our findings suggest Hum j 1 could serve as a potential serological indicator for HJ pollen-associated asthma. Nevertheless, the subgroup size of asthmatic participants without sensitization was small. Future large-scale prospective cohort studies across diverse geographic regions during *H. japonicus* pollen seasons will be critical to substantiate these findings through expanded phenotypic characterization and population-level validation. To date, several important airborne allergen proteins have been reported as marker proteins. Studies have shown that sensitization to Der p 20 or Der p 37 is associated with asthma (27, 28). A prospective case-control study reported that sensitization to profilin is a marker of severity in patients with rhinoconjunctivitis and asthma mediated by pollen (29). Moreover, researchers in China showed that sensitization to more than two allergens (e.g., Art v 1, Art v 3, and Art an 7) of *Artemisia* pollen increased the risk of allergic asthma (30). Therefore, identifying the clinical characteristics of important allergens can assist in accurate clinical diagnosis.

The development of low-IgE-reactivity peptides for allergen-specific immunotherapy (AIT) has gained momentum in recent years. For example, Bet v 1-derived contiguous overlapping peptides (COPs) induced long-term immune memory with fewer injections, offering a safer alternative to traditional AIT (31). Similarly, Huang et al. designed a hypoallergenic peptide mix containing T cell epitopes from house dust mite (HDM) allergens, which reduced IgE binding by >90% while preserving T cell reactivity (32). In the present study, heat denaturation of full-length rHum j 1 led to a certain degree of loss of IgE reactivity in the immunoblot compared with the ELISA. This could be explained by Hum j 1 being rich in cysteine, and under denaturing conditions, disulfide bond cleavage leads to the loss of major conformational epitopes. Therefore, exploring the sequential epitopes of Hum j 1 as hypoallergenic peptides is a promising immunotherapy approach. For this purpose, we synthesized six overlapping peptides (P1–P6) covering Hum j 1 to destroy IgE epitopes, with the goal to induce IgG Abs obtained through the immunization of mice and evaluate their ability to inhibit allergic patients’ IgE binding to Hum j 1. In this study, the six KLH-coupled peptides lacked relevant IgE-binding capacity and allergenic activity, and they induced strong specific IgG Abs in mice, in particular KLH-P6. Building upon the IgG-inducing capacity of KLH-conjugated peptides, subclass profiling revealed that KLH-P3, -P5, and -P6 elicited particularly robust antigen-specific IgG responses characterized by elevated IgG_1_ (Th2-associated), IgG_2a_ (Th1-associated), and IgG_2b_ (Th1/regulatory-associated) titers. This balanced IgG isotype profile suggests synergistic mechanisms combining IgE blocking (via IgG_1_) and immunomodulation (via IgG_2a_/IgG_2_b) may underlie therapeutic efficacy. Notably, this IgG isotype diversification correlated with functional superiority in downstream inhibition assays, where KLH-P6 and KLH-Pmix-induced antibodies achieved an exceptional blockade of patient-derived IgE binding. Remarkably, the mean inhibition of human IgE binding induced by mouse antibodies against KLH-P6 was 64.61%, even higher than the 57.96% inhibition induced by a mixture of six peptides (KLH-Pmix). Although KLH-P6 induced stronger blocking IgG Abs in mice and higher inhibition of allergic patients’ IgE binding to Hum j 1, KLH-Pmix may be a more comprehensive strategy, containing the full sequence of Hum j 1.

However, defining an appropriate dose of KLH-P6 in KLH-Pmix that is safer and more effective for treatment will be necessary. Peptide vaccines face challenges in clinical translation. For instance, a phase III trial of Fel d 1 peptide therapy failed to show significant clinical efficacy despite promising preclinical data (33). Although our murine model employing Freund’s adjuvant provided critical proof-of-concept evidence of IgG-mediated IgE inhibition, clinical translation necessitates a reformulation with human-compatible adjuvants. Aluminum-based adjuvants, the only adjuvants currently approved for allergy immunotherapy, preferentially induce Th2-biased responses that may complement our epitope vaccine design through distinct mechanistic pathways (34). Future studies that systematically compare the effects of aluminum adjuvants on epitope-specific IgG subclass profiles and functional neutralizing activity *in vivo* will be imperative to bridge preclinical findings to clinical applications. This underscores the importance of optimizing peptide length, epitope coverage, and adjuvant selection.

In conclusion, our findings demonstrate that rHum j 1 may enhance diagnostic accuracy by improving specificity for HJ pollen allergy while maintaining clinical relevance. Sensitization to Hum j 1 was associated with an increased risk of allergic asthma development. Furthermore, we characterized six synthetic hypoallergenic peptides derived from Hum j 1 that show promise as candidates for epitope-based AIT vaccines. While these results provide foundational insights for advancing allergy management strategies, further clinical validation is required to establish their translational applications.

## Data Availability

The original contributions presented in the study are included in the article/**Supplementary Material**. Further inquiries can be directed to the corresponding authors.

## References

[1] Frisk CA Adams-Groom B Smith M. Isolating the species element in grass pollen allergy: A review. Sci Total Environ. (2023) 883:163661. doi: 10.1016/j.scitotenv.2023.163661 37094678

[2] Brennan GL Potter C de Vere N Griffith GW Skjoth CA Osborne NJ. Temperate airborne grass pollen defined by spatio-temporal shifts in community composition. Nat Ecol Evol. (2019) 3:750–4. doi: 10.1038/s41559-019-0849-7 30962560

[3] Bernstein JA Bernstein JS Makol R Ward S. Allergic rhinitis. Jama. (2024) 331(10):866–77. doi: 10.1001/jama.2024.0530 38470381

[4] Porsbjerg C Melen E Lehtimaki L Shaw D. Asthma. Lancet. (2023) 401:858–73. doi: 10.1016/S0140-6736(22)02125-0 36682372

[5] Fukutomi Y Tanaka H Sekiya K Watai K Hamada Y Iwata M. Uncovering a severe patient group with pollen-related extrarespiratory allergic symptoms: A year-long diary survey in Japan. J Allergy Clin Immunology: In Practice. (2024) 12:1495–506.e7. doi: 10.1016/j.jaip.2024.02.011 38382879

[6] Guan K Liu B Wang M Li Z Chang C Cui L. Principles of allergen immunotherapy and its clinical application in China: contrasts and comparisons with the USA. Clin Rev Allergy Immunol. (2019) 57:128–43. doi: 10.1007/s12016-019-08751-y 31243705

[7] Ouyang YH Yin ZY Li Y Fan EZ Zhang L. Associations among air pollutants, grass pollens, and daily number of grass pollen allergen-positive patients: a longitudinal study from 2012 to 2016. Int Forum Allergy Rhinol. (2019) 9:1297–303. doi: 10.1002/alr.22389 31513736

[8] Kim JH Lim DH. Pollen of humulus japonicus: importance in South Korea. J Allergy Clin Immunol. (2016) 137(2, Supplement):AB123. doi: 10.1016/j.jaci.2015.12.533

[9] Wang Y Tan L-X Xu Z-Q Jiao Y-X Zhu D-X Yang Y-S. Identification and characterization of natural PR-1 protein as major allergen from Humulus japonicus pollen. Mol Immunol. (2023) 153:170–80. doi: 10.1016/j.molimm.2022.11.023 36525884

[10] Chen Y Wong GW Li J. Environmental exposure and genetic predisposition as risk factors for asthma in China. Allergy Asthma Immunol Res. (2016) 8:92–100. doi: 10.4168/aair.2016.8.2.92 26739401 PMC4713885

[11] Treudler R Simon JC. Overview of component resolved diagnostics. Curr Allergy Asthma Rep. (2012) 13:110–7. doi: 10.1007/s11882-012-0318-8 23076421

[12] Luo W Chen H Cheng L Cui Y Guo Y Gao Z. Chinese expert consensus on allergen component resolved diagnosis. Pediatr Allergy Immunol. (2024) 35(11):e14272. doi: 10.1111/pai.v35.11 39503267

[13] Chen K-W Zieglmayer P Zieglmayer R Lemell P Horak F Bunu CP. Selection of house dust mite–allergic patients by molecular diagnosis may enhance success of specific immunotherapy. J Allergy Clin Immunol. (2019) 143:1248–52.e12. doi: 10.1016/j.jaci.2018.10.048 30445063

[14] Hauser M Roulias A Ferreira F Egger M. Panallergens and their impact on the allergic patient. Allergy Asthma Clin Immunol. (2010) 6:1. doi: 10.1186/1710-1492-6-1 20298513 PMC2830198

[15] Park JW Ko SH Kim CW Jeoung BJ Hong CS. Identification and characterization of the major allergen of the Humulus japonicus pollen. Clin Exp Allergy. (1999) 29:1080–6. doi: 10.1046/j.1365-2222.1999.00615.x 10457112

[16] Jeong KY Han IS Choi SY Lee JS Hong CS Park JW. Allergenicity of recombinant profilins from Japanese hop, humulus japonicus. J Investig Allergol Clin Immunol. (2013) 23(5):345–50. 24260980

[17] Jeong KY Sang M Lee YS Gadermaier G Ferreira F Park J-W. Characterization of Hum j 6, a Major Allergen From Humulus japonicus Pollen, the Primary Cause of Weed Pollinosis in East Asia. Allergy Asthma Immunol Res. (2023) 15(6):767–78. doi: 10.4168/aair.2023.15.6.767 PMC10643856 37957794

[18] Gorbet MB Sefton MV. Endotoxin: the uninvited guest. Biomaterials. (2005) 26:6811–7. doi: 10.1016/j.biomaterials.2005.04.063 16019062

[19] Cheng Y-L Xu Z-Q Wang H Zhu D-X Zhu Y Sun J-L. Molecular and immunological characterization of two polcalcins as novel allergens of Artemisia sieversiana pollen. Allergology Int. (2023) 72:347–50. doi: 10.1016/j.alit.2022.10.006 36372651

[20] Holzlöhner P Hanack K. Generation of murine monoclonal antibodies by hybridoma technology. J visualized experiments: JoVE. (2017) 119(119):54832. doi: 10.3791/54832 PMC5407676 28117810

[21] Banerjee S Weber M Blatt K Swoboda I Focke-Tejkl M Valent P. Conversion of Der p 23, a New Major House Dust Mite Allergen, into a Hypoallergenic Vaccine. J Immunol. (2014) 192:4867–75. doi: 10.4049/jimmunol.1400064 PMC4582415 24733847

[22] Zabel M Weber M Kratzer B Köhler C Jahn-Schmid B Gadermaier G. Art v 1 IgE epitopes of patients and humanized mice are conformational. J Allergy Clin Immunol. (2022) 150:920–30. doi: 10.1016/j.jaci.2022.04.031 35738928

[23] Xi GP Zhang Q Yin J. Establishment and characterization of murine models of asthma and subcutaneous immunotherapy for Humulus pollen allergy. Immunity Inflammation Disease. (2021) 9:443–55. doi: 10.1002/iid3.v9.2 PMC8127558 33434413

[24] Breiteneder H Kraft D. The history and science of the major birch pollen allergen bet v 1. Biomolecules. (2023) 13(7):1151. doi: 10.3390/biom13071151 37509186 PMC10377203

[25] Zahirović A Štrukelj B Korošec P Lunder M. Epitope mapping of major ragweed allergen amb a 1. Acta Chimica Slovenica. (2019) 66(1):37–44. doi: 10.17344/acsi.2018.4516 33855488

[26] Song L-B Jiao Y-X Xu Z-Q Zhu D-X Yang Y-S Wei J-F. Identification of Pla a 7 as a novel pollen allergen group in Platanus acerifolia pollen. Int Immunopharmacol. (2023) 125(Pt A):111160. doi: 10.1016/j.intimp.2023.111160 37948987

[27] Sarzsinszky E Lupinek C Vrtala S Huang H-J Hofer G Keller W. Expression in Escherichia coli and Purification of Folded rDer p 20, the Arginine Kinase From Dermatophagoides pteronyssinus: A Possible Biomarker for Allergic Asthma. Allergy Asthma Immunol Res. (2021) 13(1):154–63. doi: 10.4168/aair.2021.13.1.154 PMC7680834 33191683

[28] Huang H-J Resch-Marat Y Casset A Weghofer M Zieglmayer P Zieglmayer R. IgE recognition of the house dust mite allergen Der p 37 is associated with asthma. J Allergy Clin Immunol. (2022) 149:1031–43. doi: 10.1016/j.jaci.2021.07.040 34419535

[29] RuizuHornillos J L,rnillos3) MA Berges Jimeno P Henrnolos A Blanco S Seoane-eoanelos3 M. Profilin is a marker of severity in allergic respiratory diseases. Allergy. (2020) 75:853–61. doi: 10.1111/all.14140 31804710

[30] Gao ZS Fu WY Sun YM Gao BY Wang HY Liu ML. Artemisia pollen allergy in China: Component-resolved diagnosis reveals allergic asthma patients have significant multiple allergen sensitization. Allergy. (2019) 74:284–93. doi: 10.1111/all.2019.74.issue-2 PMC6587742 30155917

[31] Spertini F Perrin Y Audran R Pellaton C Boudousquié C Barbier N. Safety and immunogenicity of immunotherapy with Bet v 1–derived contiguous overlapping peptides. J Allergy Clin Immunol. (2014) 134:239–40.e13. doi: 10.1016/j.jaci.2014.04.001 24797422

[32] Huang H-J Curin M Banerjee S Chen K-W. A hypoallergenic peptide mix containing T cell epitopes of the clinically relevant house dust mite allergens. Allergy. (2019) 74:2461–78. doi: 10.1111/all.v74.12 PMC7078969 31228873

[33] Wraith DC Krishna MT. Peptide allergen-specific immunotherapy for allergic airway diseases-State of the art. Clin Exp Allergy. (2021) 51:751–69. doi: 10.1111/cea.13840 33529435

[34] Lan J Feng D He X Zhang Q Zhang R. Basic properties and development status of aluminum adjuvants used for vaccines. Vaccines. (2024) 12(10):1187. doi: 10.3390/vaccines12101187 39460352 PMC11511158

