# Supplementary material for: Characterization of the clinical relevance and hypoallergenic peptides of the newly evidenced major allergen Hum j 1
Source: Front Immunol. 2025 Jun 23;16:1588870. doi: 10.3389/fimmu.2025.1588870 (PMC12230049; doi:10.3389/fimmu.2025.1588870)
Supplement: Supplementary file 1 [file Table1.docx]

**Supplementary materials**

**Supplementary Table S1** Clinical characteristics, SPT response and IgE-reactivity of the *Humulus japonicus* allergic patients

| **No.** | **Gender** ^a^ | **Age** | **Diag.** ^b^ | **Seasons of symptoms** ^c^ | | **Skin test** | **w22-sIgE(grade)** |
| --- | --- | --- | --- | --- | --- | --- | --- |
| 1 | 1 | 5 | AD; R; A | | 1,3,4 | No | >100(6) |
| 2 | 2 | 37 | R; C; Cough; Wheeze | | 3 | +++ | >100(6) |
| 3 | 1 | 12 | R; C; A | | 1,3 | ++++ | >100(6) |
| 4 | 2 | 27 | R; C | | 1,3 | ++++ | >100(6) |
| 5 | 2 | 62 | A; R; C | | 1,3 | No | 81.8(5) |
| 6 | 1 | 8 | AD; R | | 5 | No | 76.9(5) |
| 7 | 1 | 13 | R; AD | | 5 | +++ | 71.1(5) |
| 8 | 2 | 12 | R; A | | 1,3 | ++++ | 69.6(5) |
| 9 | 1 | 17 | R | | 5 | No | 43.6(4) |
| 10 | 2 | 5 | R; C; Cough | | 5 | +++ | 43.2(4) |
| 11 | 2 | 33 | R; C; A | | 3 | No | 37.2(4) |
| 12 | 2 | 8 | R; A; C | | 3 | No | 37.2(4) |
| 13 | 1 | 5 | R; C | | 5(3) | +++ | 7.63(3) |
| 14 | 2 | 38 | R; A; AD; C | | 1,3 | No | 17.1(3) |
| 15 | 2 | 42 | R; C | | 1,3 | ++++ | 12.2(3) |
| 16 | 1 | 18 | A; R; C | | 1,3 | No | 12.1(3) |
| 17 | 1 | 32 | U; R | | 3 | No | 2.84(2) |
| 18 | 1 | 7 | R; U; Eczema | | 5 | ++ | 2.52(2) |
| 19 | 2 | 51 | R; A; C | | 3 | + | 2.49(2) |
| 20 | 1 | 32 | R; Food allergy | | 3 | No | 1.98(2) |
| 21 | 2 | 48 | R; A; C | | 1,3 | +++ | 14.7(3) |
| 22 | 2 | 23 | R; C; A | | 5 | ++ | 24.4(4) |
| 23 | 2 | 48 | R; C; Dermatitis | | 1,3 | +++ | 15.5(3) |
| 24 | 2 | 39 | R; A | | 5(3) | ++++ | 13.2(3) |
| 25 | 1 | 11 | R; A; C | | 1,3 | +++ | 96.7(5) |
| 26 | 1 | 35 | R; Eczema | | 5(1,3) | No | 6.82(3) |
| 27 | 2 | 17 | R; C; U; Eczema | | 1,3 | No | 73.4(5) |
| 28 | 1 | 11 | R; C | | 5(1,3) | +++ | 31.3(4) |
| 29 | 1 | 46 | R; C | | 1,3 | No | 25.7(4) |
| 30 | 2 | 48 | R; A; C; Dermatitis | | 3 | ++++ | 6.69(3) |
| 31 | 1 | 3 | R; AD | | 3,4 | No | 9.39(3) |
| 32 | 2 | 59 | Eyelid edema; R; A; C | | 5(3) | No | 30.2(4) |
| 33 | 1 | 8 | R; C | | 5(1,3) | +++ | 46.4(4) |
| 34 | 1 | 29 | R; A; C | | 5(3) | ++ | 8.42(3) |
| 35 | 2 | 28 | R; C | | 1,3 | No | 8.64(3) |
| 36 | 1 | 53 | Dermatitis; A; R | | 1,3 | +++ | 7.29(3) |
| 37 | 2 | 48 | R; A; C; U | | 3 | ++ | 14.4(3) |
| 38 | 2 | 49 | Cough; R; C | | 5(1,3) | +++ | 8.38(3) |
| 39 | 2 | 50 | R; Cough; C; Eczema | | 3 | +++ | 8.12(3) |
| 40 | 2 | 38 | R; C | | 1,3 | No | 33.3(4) |
| 41 | 2 | 12 | R; A | | 5(3) | No | 40.7(4) |
| 42 | 2 | 41 | R; A; C; U | | 1,3 | ++++ | 10.2(3) |
| 43 | 2 | 37 | R; C; AD | | 1,3 | ++ | 16.5(3) |
| 44 | 2 | 13 | Dermatitis; R | | 5(1,3) | ++++ | 64.9(5) |
| 45 | 2 | 43 | R | | 5(3) | ++ | 1.22(2) |
| 46 | 1 | 37 | R | | 1,3 | ++ | 3.95(3) |
| 47 | 1 | 36 | Anaphylaxis; R | | 5(1,3) | No | 52.9(5) |
| 48 | 2 | 17 | R; C | | 1,3 | No | 8.12(3) |
| 49 | 2 | 15 | A; R | | 5 | No | 20.4(4) |
| 50 | 2 | 3 | R; C | | 1,3 | No | 7.74(3) |
| 51 | 2 | 12 | R | | 5(1,3) | ++ | 32(4) |
| 52 | 1 | 38 | R; A | | 1,3 | No | 28.4(4) |
| 53 | 1 | 32 | R; A; C | | 1,3 | ++++ | 18.5(4) |
| 54 | 1 | 28 | R; A; Eczema | | 1,3 | + | 20(4) |
| 55 | 1 | 8 | Cough; R; C | | 5(4) | +++ | 4.86(3) |
| 56 | 1 | 40 | R; A; C | | 5(1) | No | 66(5) |
| 57 | 1 | 7 | R | | 5(1,3) | + | 4.36(3) |
| 58 | 1 | 11 | R | | 1,3 | No | 9.38(3) |
| 59 | 2 | 40 | R; C | | 1,3 | No | 9.48(3) |
| 60 | 1 | 28 | R; C | | 1,3 | ++ | 15.8(3) |
| 61 | 1 | 8 | R; A; C | | 5(1,3) | No | 7.89(3) |
| 62 | 1 | 11 | R; C; Cough | | 1,3 | ++ | 16(3) |
| 63 | 2 | 41 | R; C; A | | 1,3 | ++ | 4.91(3) |
| 64 | 2 | 39 | R; A; C | | 1,3 | No | 8.11(3) |
| 65 | 2 | 42 | R; C | | 1,3 | No | 18.9(4) |
| 66 | 1 | 32 | R; C; A | | 1,3 | +++ | 14.3(3) |
| 67 | 2 | 28 | Dermatitis; R; C | | 3 | No | 47.3(4) |
| 68 | 1 | 55 | R; C | | 1,3 | No | 3.54(3) |
| 69 | 1 | 44 | R; C; A | | 1,3 | No | 18.6(4) |
| 70 | 1 | 17 | R; C | | 1,3 | No | 57.4(5) |
| 71 | 2 | 53 | R; A; C; U | | 5(3) | ++++ | 28.4(4) |
| 72 | 2 | 12 | R | | 1,3 | No | >100(6) |
| 73 | 2 | 38 | U; R; C | | 1,3 | No | 79.2(5) |
| 74 | 2 | 49 | AD; R; A | | 1,3 | No | >100(6) |
| 75 | 1 | 15 | R; C | | 1,3 | No | >100(6) |
| 76 | 2 | 39 | R; C | | 1,3 | No | 1.46(2) |
| 77 | 2 | 11 | R; C | | 5(1,3) | ++++ | 83.7(5) |
| 78 | 2 | 11 | R; C | | 1,3 | No | 57.6(5) |
| 79 | 2 | 41 | R; C | | 1,3 | ++ | 14.7(3) |
| 80 | 1 | 35 | Drug allergy; Dermatitis; R | | 3 | No | 7.27(3) |
| 81 | 2 | 38 | R; C | | 1,3 | No | 15.5(3) |
| 82 | 2 | 38 | R; C; U | | 1,3 | No | 1.26(2) |
| 83 | 1 | 10 | R; C | | 1,3 | No | >100(6) |
| 84 | 2 | 45 | R; A; C | | 1,3 | No | 10.7(3) |
| 85 | 1 | 38 | R; C | | 1,3 | ++ | 24.1(4) |
| 86 | 2 | 41 | R; A; C | | 3 | ++ | >100(6) |
| 87 | 1 | 43 | R; C | | 1,3 | No | 21.4(4) |
| 88 | 1 | 9 | R; A | | 5(1,3) | +++ | 55.8(5) |
| 89 | 2 | 31 | U; R | | 5 | No | 24.1(4) |
| 90 | 1 | 7 | R | | 5 | No | >100(6) |
| 91 | 1 | 47 | R; C; Dermatitis | | 1,3 | No | 3.05(2) |
| 92 | 1 | 11 | U; R | | 1,3,4 | ++++ | 38.5(4) |
| 93 | 2 | 8 | R; C; A | | 5(1,3) | + | 66.2(5) |

^a^1: male; 2: Female.

^b^ AD, Atopic dermatitis; R, Allergic rhinitis; A, Asthma; C, Allergic rhinoconjunctivitis; U, Urticaria.

^c^1: spring, 2: summer, 3: autumn, 4: winter, 5: during year.

**Supplementary Table S2.** Characteristics of Hum j 1-derived synthetic peptides

|  | Position aa | Sequence | Number of aa | Molecular weight | Isoelectric point(pI) |
| --- | --- | --- | --- | --- | --- |
| P1 | 1-22 | DNCFENGMKACTSLYDKYYQNC | 22 | 2610.9 | 4.31 |
| P2 | 11-33 | CTSLYDKYYQNCVMKLPPGACID | 23 | 2626.1 | 6.13 |
| P3 | 22-44 | CVMKLPPGACIDSENYRKCLTNH | 23 | 2593.0 | 8.01 |
| P4 | 33-53 | DSENYRKCLTNHIGSCDIDTC | 21 | 2387.6 | 4.44 |
| P5 | 48-70 | CDIDTCFEDVSIACRSIYPSNYA | 23 | 2585.8 | 3.5 |
| P6 | 61-85 | CRSIYPSNYAECATTHHNICGDLQG | 25 | 2754.0 | 6.02 |


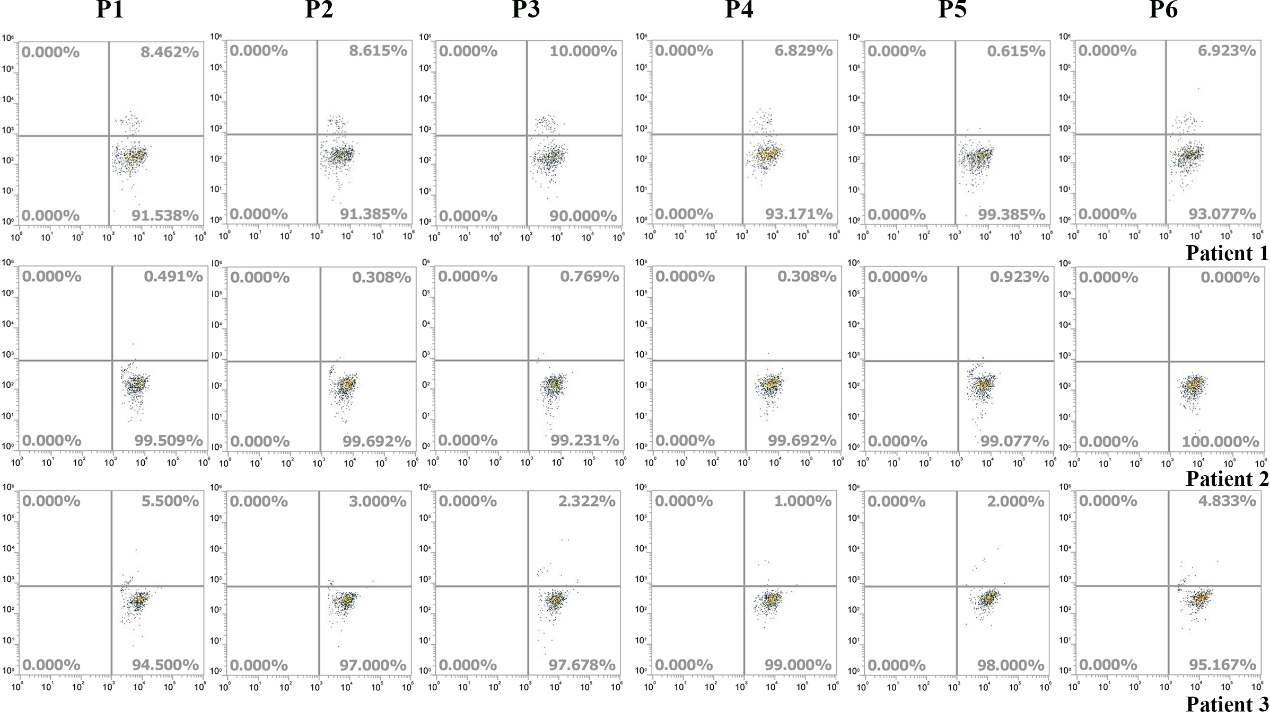


**Supplementary Fig S1.** Allergenic activity of Hum j 1 derived KLH-peptides. Representative flow cytometry plots of basophils from 3 different *Humulus japonicus* allergic patients and stimulated with Hum j 1 derived KLH-peptides (P1 to P6), respectively. Basophil activation was assessed by monitoring the proportion of CCR3 and CD63 positive cells.


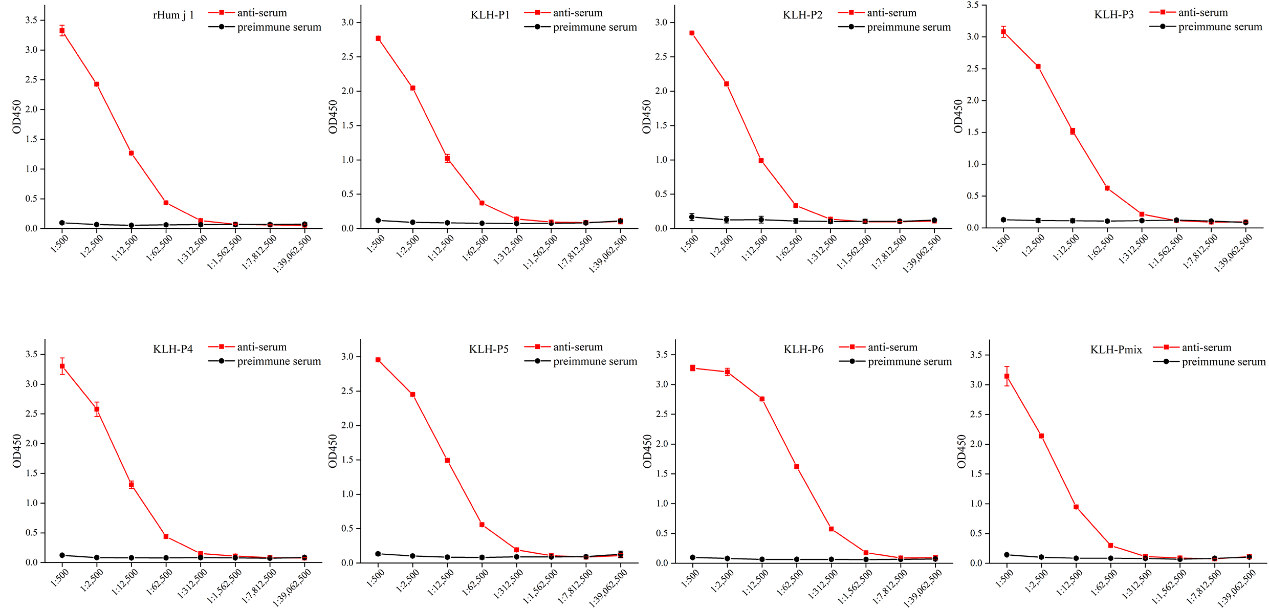


**Supplementary Fig S2.** Hum j 1–speciﬁc IgG responses of mouse anti–recombinant Hum j 1 and anti–KLH-coupled peptides antisera. Hum j 1–speciﬁc IgG levels (y-axes: OD values) of different dilutions (1:500–1:39,062,500) (x-axes) of antisera from mouse that were immunized with recombinant Hum j 1, KLH-P1, KLH-P2, KLH-P3, KLH-P4, KLH-P5, KLH-P6, or KLH-Pmix adsorbed to CFA are shown in comparison with

the corresponding preimmune sera. Results are displayed as mean values ± SD of three determinations performed with sera from three mice.


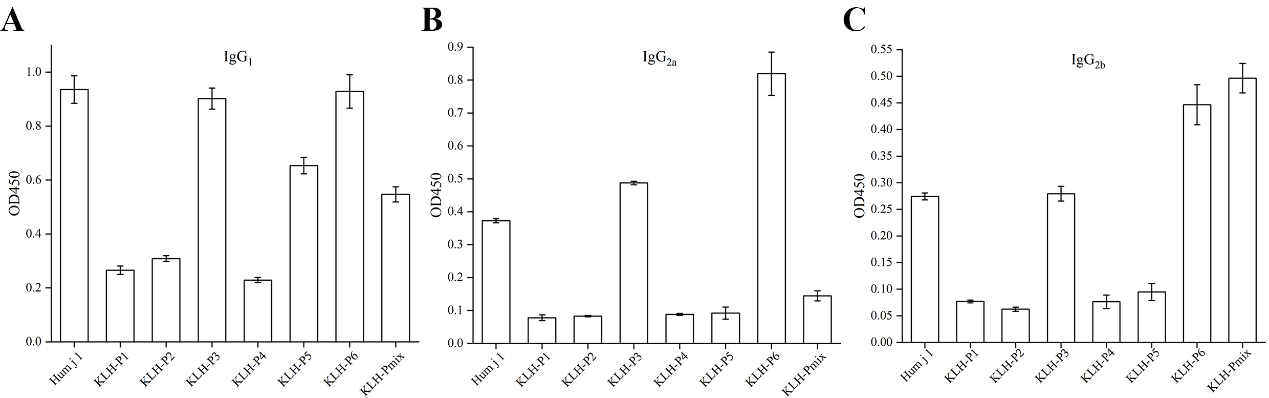


**Supplementary Fig S3.** IgG_1_, IgG_2a_ and IgG_2b_-recognition of Hum j 1 and Hum j 1 peptides. Serum levels (optical densities, ODs) (y-axes) of Hum j 1-and peptides-specific IgG_1_ (A), IgG_2a_ (B) and IgG_2b_ (C) (x-axes) are shown as bar charts. Results are displayed as mean values ± SD of three determinations performed with sera from three mice.

**Supplementary Table S3** Inhibition of patients’ IgE binding to Hum j 1 with mouse IS to Hum j 1 and Hum j 1-derived peptides.

|  | Mouse IS | | | | | | | |
| --- | --- | --- | --- | --- | --- | --- | --- | --- |
| No. | Anti-Hum j 1 | Anti-KLH-P1 | Anti-KLH-P2 | Anti-KLH-P3 | Anti-KLH-P4 | Anti-KLH-P5 | Anti-KLH-P6 | Anti-KLH-Pmix |
| 7 | 72.20 | 4.26 | 13.74 | 29.50 | 19.38 | 18.66 | 52.31 | 39.54 |
| 10 | 83.86 | 10.71 | 16.35 | 23.64 | 16.57 | 18.90 | 46.70 | 45.25 |
| 11 | 89.50 | 14.16 | 22.69 | 37.65 | 27.29 | 25.54 | 62.57 | 46.64 |
| 15 | 89.10 | 19.48 | 31.89 | 42.07 | 34.36 | 33.13 | 62.59 | 56.29 |
| 21 | 89.60 | 20.25 | 25.89 | 42.77 | 32.98 | 32.16 | 59.76 | 56.84 |
| 22 | 84.68 | 22.37 | 29.71 | 42.06 | 34.84 | 39.56 | 61.93 | 56.35 |
| 26 | 89.06 | 34.08 | 40.92 | 58.64 | 43.66 | 47.65 | 67.49 | 63.58 |
| 30 | 87.39 | 27.98 | 38.31 | 52.79 | 37.05 | 43.27 | 64.29 | 65.56 |
| 32 | 81.60 | 12.94 | 22.77 | 36.55 | 24.13 | 30.78 | 49.68 | 50.79 |
| 34 | 82.43 | 33.84 | 35.61 | 50.33 | 42.75 | 43.28 | 66.38 | 63.82 |
| 38 | 88.74 | 22.44 | 31.45 | 47.18 | 36.40 | 38.01 | 70.00 | 62.02 |
| 42 | 91.29 | 27.30 | 31.39 | 47.63 | 41.39 | 41.18 | 75.13 | 60.56 |
| 44 | 84.67 | 59.21 | 59.83 | 69.31 | 60.85 | 60.33 | 74.07 | 72.66 |
| 46 | 77.80 | 25.25 | 32.41 | 48.37 | 39.35 | 43.47 | 68.15 | 65.57 |
| 49 | 89.60 | 13.40 | 25.34 | 36.47 | 28.48 | 31.86 | 62.46 | 53.96 |
| 50 | 89.04 | 27.12 | 30.78 | 49.15 | 37.92 | 43.62 | 70.20 | 65.10 |
| 51 | 82.41 | 32.18 | 36.50 | 54.14 | 44.93 | 51.62 | 81.67 | 74.44 |
| 52 | 89.64 | 23.54 | 30.82 | 44.39 | 38.95 | 40.71 | 73.19 | 59.82 |
| 53 | 86.96 | 11.85 | 23.97 | 38.27 | 25.90 | 28.04 | 61.40 | 50.97 |
| 56 | 76.77 | 3.17 | 13.10 | 17.06 | 12.21 | 14.49 | 40.72 | 35.59 |
| 58 | 79.44 | 24.57 | 28.89 | 47.32 | 43.11 | 45.32 | 73.64 | 67.70 |
| 60 | 89.12 | 12.05 | 24.46 | 34.72 | 29.01 | 35.31 | 65.79 | 53.26 |
| 63 | 91.31 | 22.85 | 29.08 | 47.25 | 37.42 | 36.13 | 77.65 | 62.53 |
| 64 | 85.73 | 25.24 | 33.87 | 44.12 | 42.99 | 44.28 | 62.77 | 62.12 |
| **Mean** | **85.50** | **22.09** | **29.57** | **43.39** | **34.66** | **36.97** | **64.61** | **57.96** |
